# Focal therapy using magnetic resonance image-guided focused ultrasound in patients with localized prostate cancer

**DOI:** 10.1186/s40349-016-0054-y

**Published:** 2016-03-11

**Authors:** Bertram Yuh, An Liu, Robert Beatty, Alexander Jung, Jeffrey Y. C. Wong

**Affiliations:** Division of Urology, City of Hope National Medical Center, 1500 East Duarte Road, Duarte, CA 91010 USA; Department of Radiation Oncology, City of Hope National Medical Center, 1500 East Duarte Road, Duarte, CA 91010 USA; Department of Diagnostic Radiology, City of Hope National Medical Center, 1500 East Duarte Road, Duarte, CA 91010 USA

**Keywords:** Focused ultrasound, Focal therapy, Magnetic resonance image-guided focused ultrasound, Prostate cancer

## Abstract

**Background:**

The purpose of this study is to evaluate and report the feasibility, safety, and initial outcomes of patients with limited localized prostate cancer treated using a trans-rectal magnetic resonance image-guided focused ultrasound (MRGFUS) device. Attempts to focally treat only the index lesion for prostate cancer have been explored to reduce side effects while maintaining oncologic control. MRGFUS allows for precise targeting of thermal ablative therapy with real-time thermometry.

**Methods:**

Three patients underwent multiparametric 3T MRI and TRUS-guided 16-sector mapping biopsies of the prostate. The patients were eligible if they had Gleason 6 or 7 (3 + 4) disease, no MRI-visible tumor ≥15 mm, no extracapsular extension, and no more than two discrete cancerous lesions ≤10 mm in length. Acoustic power was adjusted to achieve temperatures of 65 to 85 °C.

**Results:**

Age ranged from 60 to 64 years. The number of biopsy-positive sectors treated ranged from 2 to 4. Post therapy, 16-sector biopsies at 6 months were negative in two patients with one patient still with Gleason 6 cancer (10 %, 2 mm) in one core. 16-sector biopsy in the first patient remains negative at 24 months. PSA continues to remain stable in all patients. IPSS in all patients either remained stable or decreased then stabilized. Erectile function according to the International Index of Erectile Function (IIEF) was excellent for all patients and demonstrated no decline up to the time of last follow-up at 12–24 months.

**Conclusions:**

MRGFUS is a feasible alternative for focal therapy in a select subset of patients with prostate cancer. The treatment is well tolerated with no evidence of decline in functional outcomes. Initial post-therapy biopsy results are promising. Long-term treatment efficacy requires further study.

## Background

Prostate cancer is the most common non-cutaneous malignancy diagnosed in men. As more than 60 % of these cancers are considered multifocal, the standard of care treatment for localized disease is whole gland therapy [1]. Unfortunately, the most common of these treatments, surgery or radiation therapy, can be associated with long-term functional side effects [2]. Recently, attempts to treat the index lesion as part of a focal therapy strategy have been explored to reduce side effects while maintaining adequate oncologic control. Treatment modalities that have been evaluated include cryotherapy, laser therapy, high-intensity focused ultrasound (HIFU), photodynamic therapy, and brachytherapy.

The experience with HIFU for focal therapy has been limited and has primarily utilized ultrasound imaging to target therapy. Marien et al. reviewed the published focal therapy experience using HIFU which consisted of six studies and a total of 133 patients [3]. Urinary retention and dysuria ranged from 10 to 28 %, erectile dysfunction from 20 to 50 %, and urinary incontinence from 0 to 10 %. Biopsy-proven recurrence rates varied from 1 to 20 % for the entire prostate gland and 1 to 10 % for the treated lobe. Ahmed et al. recently updated their experience and reported on 56 men with multifocal localized prostate cancer treated to the index lesion using HIFU [4]. The pad-free and leak-free continence rate was 92.3 %, and erections sufficient for intercourse were preserved in 76.9 %. Histopathologic absence of clinically significant cancer on the treated side was observed in 84.6 % and for the entire gland 80.8 %. Finally, Cordeiro Feijoo et al. reported on 67 men who underwent HIFU hemiablation [5]. Negative biopsies of the treated lobe were achieved in 83.6 % of the patients.

Multiparametric magnetic resonance imaging (MRI) has become more prevalent in the staging and targeted biopsy of prostate cancer. Recently, magnetic resonance image-guided focused ultrasound (MRGFUS) has combined MRI imaging and HIFU to allow for precise tumor targeting and thermometry with real-time temperature feedback within the gland during treatment. Napoli et al. showed that in patients treated with MRGFUS followed by radical prostatectomy, the prostate specimen demonstrated accurate coagulative necrosis in the treatment zone indicative of good energy delivery [6]. Lindner et al. previously reported on one case using MRGFUS though oncologic outcome was not examined [7]. This experience was recently updated by Ghai et al. [8]. Four patients with cT1c–T2a Gleason 6 very low risk and low risk disease were treated. Three were biopsy negative post therapy, representing complete ablation of five of the six target lesions. All patients had at least one Gleason 6-positive core outside the treated zone. Quality of life scores were unchanged 3 months after therapy. Herein, we report the feasibility, safety, and initial post-therapy outcomes of the first three patients with prostate cancer treated in the USA using a trans-rectal MRGFUS device.

## Methods

From November 2013 to July 2014, three men (ages 60–64) met eligibility criteria and received MRGFUS focal therapy for prostate cancer as part of an Institutional Review Board (IRB)-approved trial sponsored by Insightec, Inc. (Haifa, Israel). Inclusion criteria included (1) age between 55 and 75 years, (2) PSA <10, (3) Gleason score ≤7 (3 + 4), (4) cT1c and cT2a, (5) maximum of two lesions on most recent mapping biopsy with each cancer site <10 mm in length, (6) prostate gland size <60 ml, (7) no prior TURP, and (8) PSA density <0.15. Patients treated with medications that could affect PSA value were eligible if therapies were discontinued at least 3 months prior to study entry.

**Fig. 1:**
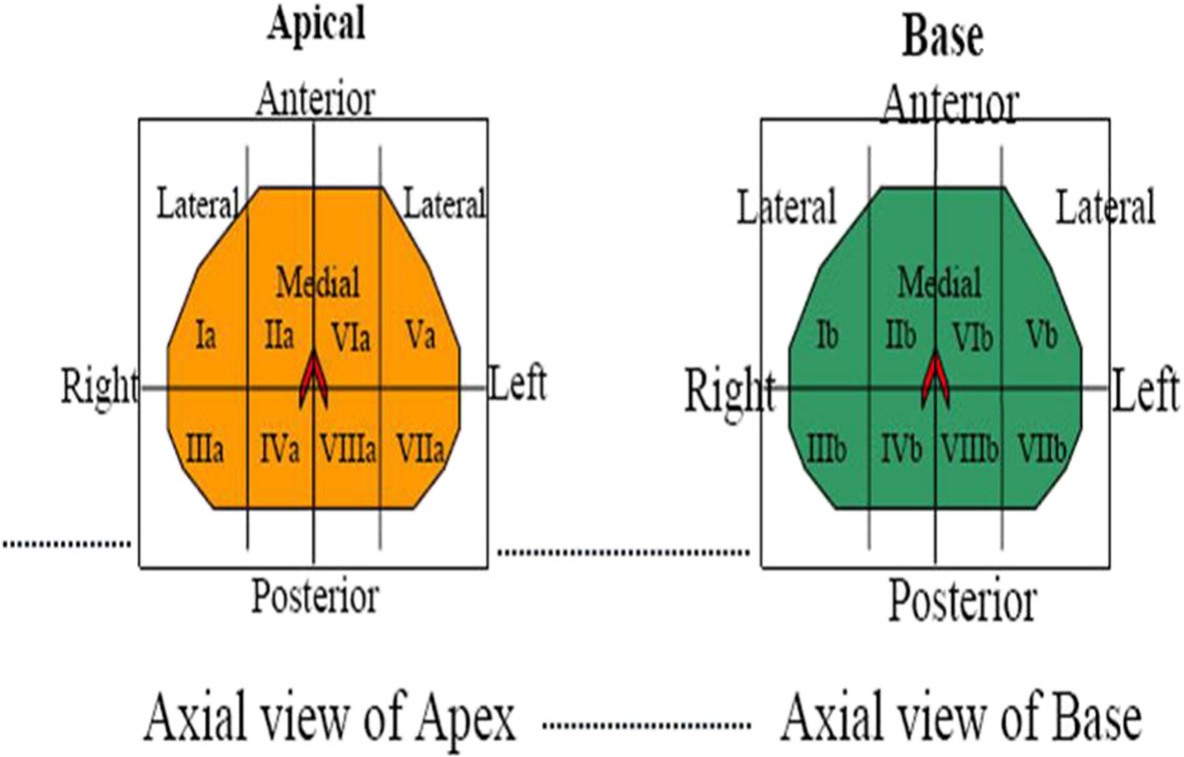
Biopsy mapping template. Biopsy sectors were designated as follows: right anterior lateral apical (*Ia*); right anterior medial apical (*IIa*); right posterior lateral apical (*IIIa*); right posterior medial apical (*IVa*); left anterior lateral apical (*Va*); left anterior medial apical (*VIa*); left posterior lateral apical (*VIIa*); left posterior medial apical (*VIIIa*); right anterior lateral base (*Ib*); right anterior medial base (*IIb*); right posterior lateral base (*IIIb*); right posterior medial base (*IVb*); left anterior lateral base (*Vb*); left anterior medial base (*VIb*); left posterior lateral base (*VIIb*); left posterior medial base (*VIIIb*)

All men underwent imaging with multiparametric 3T MRI (GE Signa HDxt) without endorectal coil and CT (GE Optima 560 PET-CT) and completed functional assessment of urinary symptoms and erectile function using validated questionnaires (International Prostate Symptom Score (IPSS) and International Index of Erectile Function (IIEF)). MRI parameters are presented in Table 1. Patients were eligible for therapy if any MRI-visible tumor measured <15 mm, if no extracapsular extension was demonstrated, and if no calcifications were identified on CT that would be in the beam’s path. A trans-rectal ultrasound (TRUS)-guided mapping biopsy of the prostate was then performed by the urologist of the treatment team (BY) similar to a brachytherapy template with core biopsies of 16 sectors (Fig. 1). Patients were eligible for therapy if no more than two discrete cancerous lesions were identified, if ≤4 sectors were positive for cancer (i.e., two for each cancerous lesion), if the total length of cancer focus was <10 mm, and if each positive core was <7 mm in length.

**Table 1:** MRI parameters

Pulse sequence name	Axial T1 FSE	Axial/sag/coronal T2 FRFSE			Axial DWI echo planar imaging (EPI)			Axial DCE GRE/FSPGR	Pre-DCE T1 maps		
Plane(s) of acquisition	Obl/Axial	oAx	oSag	oCor	oAx			Ax	Ax	Ax	Ax
TR (msec)	750	2520	3050	2830	4275			2.5	2.5	2.5	2.5
TE (msec)	7.0	117	102	102	80			0.8	0.8	0.8	0.8
Flip angle	90	90	90	90	90			12	15	10	5
Matrix (cm)	256/192	320/256	320/256	320/256	128/128			160/160	160/160	160/160	160/160
Field of view (cm)	38/30	22/22	18/18	18/18	38/38			22/22	22/22	22/22	22/22
Slice thickness and slice gap (mm)	5.0/1.0	3.0/0.5	3.0/0.5	3.0/0.5	3.0/0.5			5.0/0.0	5.0/0.0	5.0/0.0	5.0/0.0
Number of signal averages	3	4	3	3	2	3	3	1.1	2.0	2.0	2.0
Parallel imaging factor					TETRA/asset						
Presence of fat suppression	None	None	None	None	Yes			None	None	None	None
3 B values (s/mm^2^)	None	None	None	None	100	800	1400	None	None	None	None

Vendor: GE 3T HDxt 16.0. Field strength 3 T. Type of coil (number of channels): HD TORSO ARRAY 8 CH. No endorectal coil. For DCE: concentration of gadolinium (use 0.1 mmol/kg) MultiHance (gadobenate dimeglumine); rate of power injection 3 ml/s/volume based on weight of patient; volume of saline flush 3 ml/s/20 ml saline flush; injection and scan delay: imaging begins/acquired immediately. After 22 s of imaging, gadolinium is injected (22-s contrast injection scan delay); time per phase 8 s per phase/total scan time 04:33; number of phases 35

*EPI* echo planar imaging, *FRFSE* fast-relaxation fast spin echo, *FSE* fast spin echo, *GRE* gradient echo, *DCE* dynamic contrast exam, *FSPGR* fast spoiled gradient echo, *DWI* diffusion-weighted imaging

**Fig. 2:**
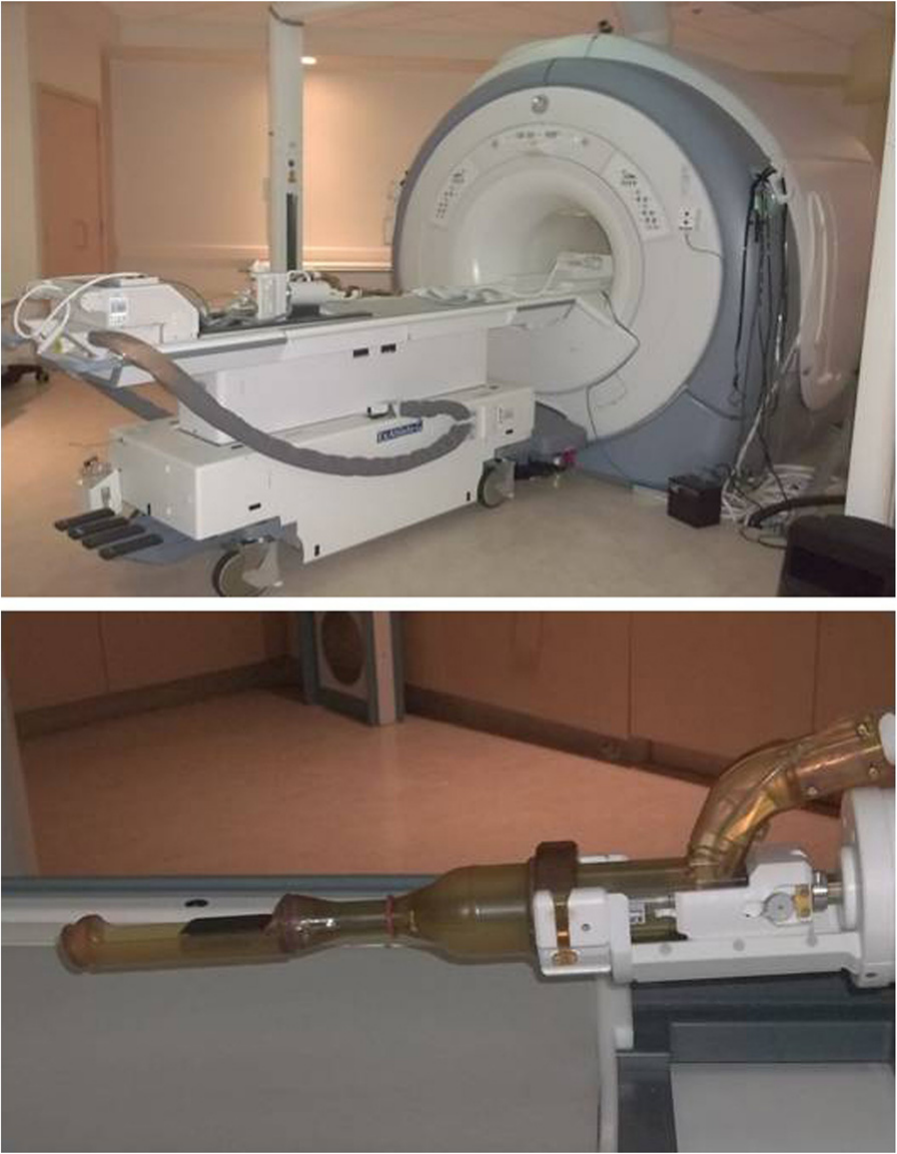
ExAblate 2100 treatment table docked to the MRI scanner (*top*). Trans-rectal ultrasound transducer used to deliver therapy (*bottom*) which consists of a 990-element phased array transducer covered by an endorectal balloon containing circulating chilled (14 °C degassed water)

Three men met all eligibility criteria and received MRGFUS therapy 1 month after biopsy. All treatments were administered using the ExAblate 2100 MRGFUS system (Insightec Inc, Haifa, Israel) (Fig. 2) and were performed as an outpatient under general anesthesia by the same treatment team in a dedicated MRI suite in the Department of Radiation Oncology. Prior to the day of the procedure, the patient’s MRI and CT scans were loaded into an Eclipse radiation oncology treatment planning system (Varian, Inc., Palo Alto, CA). The prostate gland was contoured, and results from the 16-sector mapping biopsy were used to contour the sectors of the gland to be treated. Normal organ avoidance structures (neurovascular bundles, urethra, bladder wall, and rectal wall) were also contoured.

Patients were placed on a low-residue diet 48 h prior, a standard colonoscopy prep procedure started the day prior, and a cleansing enema was administered 2 h prior to the procedure. In the morning of the procedure, a QA sonication was performed on a phantom gel to verify the geometrical accuracy of treatment delivery. The patient was then brought into the treatment suite, general anesthesia was induced, a Foley catheter was placed, and the patient was transferred to the ExAblate 2100 system table and positioned supine in a knee-bent position. The ExAblate 2100 trans-rectal FUS transducer was then carefully positioned in the rectum, and the patient moved into the 3T MRI scanner (GE HDX). An endorectal balloon covered the FUS transducer and was constantly filled with circulating chilled degassed water to cool the rectal wall during treatment delivery. T2-weighted images verified appropriate positioning of the transducer relative to the prostate gland. T1-weighted images confirmed acoustic coupling of the endorectal balloon with the rectal wall.

The prostate gland, sectors to be treated, neurovascular bundles, urethra, anterior rectal wall, and bladder wall were then identified and contoured on each axial MRI image. As per protocol, since none of the biopsy-positive regions were visible on MRI, therapy was directed to the regions or sectors which were biopsy positive as defined by the biopsy template (Fig. 1). The ExAblate 2100 system then determined the number of sonications needed to treat and the proposed beam paths on each axial slice. With each sonication, the treating physician first reviewed and approved the proposed beam path and acoustic energy to deliver. MRI thermometry after each sonication verified that the actual temperature approximated what was predicted per treatment plan. Acoustic power was adjusted to achieve temperatures in the range of 65 to 85 °C. At the completion of the treatment procedure, a final MRI scan was performed and T2-weighted and T1 contrast-enhanced sequences obtained to determine the volume of non-perfused prostate gland volume that was present as a result of therapy. Patients were then transported to a postsurgical recovery area, the Foley discontinued, and the patient discharged home on antibiotics and an alpha-blocker per standard institutional post prostate permanent seed brachytherapy protocol.

Pain was measured on a 10-point numeric scale postoperatively. Follow-up of patients post therapy included assessment per protocol at 1 week and 1, 3, 6, 9, 12, and 18 months, with repeat 16-sector mapping biopsies at 6 months. Questionnaires were completed in person during follow-up visits. IIEF-15 scores for the erectile dysfunction domain (questions 1–5, 15) as well as total questionnaire scores were tabulated.

## Results

Baseline demographic and treatment characteristics of the three patients treated are listed in Table 2. Age ranged from 60 to 64 years. No cancers were visible by CT or MRI, and prostate volume ranged from 21 to 51 ml. Two patients had no prior therapy. Patient 3 received a 3-month injection of leuprolide 9 months prior to therapy. The number of sectors which were biopsy positive and treated ranged from 2 to 4. Patient 1 had bilateral disease.

**Table 2:** Patient pre-therapy characteristics and treatment details

	Patient 1	Patient 2	Patient 3
Age	64	60	61
BMI	26.5	25.4	23.9
Prostate volume (ml)	45.6	51	21
Gleason score	3 + 3	3 + 3	3 + 3
Clinical stage	T1c	T1c	T1c
Pre-therapy PSA (ng/ml)	1.96	4.13	2.14
Previous therapy	None	None	Leuprolide 3-month duration (stopped 6 months prior to protocol entry)
Pre-therapy multiparametric MRI and CT	No visible lesion	No visible lesion	No visible lesion
Sectors positive on biopsy pre-therapy (% and length of core)	Right posterior lateral base (2 %, 0.2 mm) Left lateral base (5 %, 0.5 mm)	Left anterior lateral apex (1 %, 0.2 mm) Left anterior medial apex (1 %, 0.5 mm) Left posterior lateral apex (10 %, 5 mm)	Left anterior medial apex (10 %, 2 mm) Left posterior lateral apex (5 %, 1 mm) Left posterior medial apex (20 %, 2.5 mm) Left posterior medial base (10 %, 2 mm)
Number of treatment (macro) sonications	11	6	12
Total sonication time	3 h 55 min	1 h 3 min	1 h 46 min
Total time in scanner	6 h 18 min	3 h 34 min	3 h 40 min
Pain score postoperatively	0–1	0–1	0–1

**Fig. 3:**
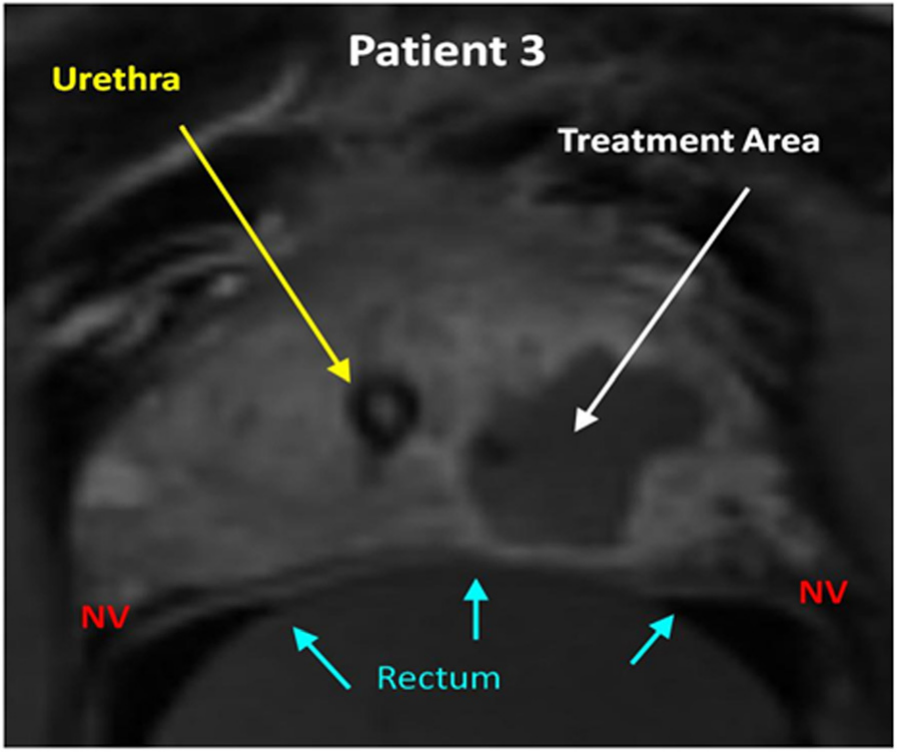
Post contrast T1-weighted axial image immediately after the completion of therapy in patient 3 with Gleason 6 disease in the left anterior medial apex, left posterior lateral apex, left posterior medial apex, and left posterior medial base. The treatment area demonstrates non-perfusion of tissue after MRGFUS thermal ablation. *NV* neurovascular bundle

Treatment time for patient 1 was 3 h 55 min and significantly longer than for patients 2 and 3. This was due to an ExAblate system hardware fault which was corrected after approximately 30 min and to movement of the patient midway through the session requiring repositioning. Subsequent patients were treated with the addition of paralytics to general anesthesia with treatment times reduced to less than 2 h. The total number of treatment macro-sonications ranged from 6 to 12. Immediately after the final treatment sonication, all patients underwent a post-therapy MRI scan with and without contrast while still in the treatment position to correlate areas of non-perfusion within the gland with areas which were intended to be treated. Figure 3 demonstrates the area of non-perfusion post therapy in patient 3. No patient demonstrated intra-prostatic hemorrhage on post-therapy MRI imaging. All men tolerated the procedure well and were discharged home the same day. The Foley catheter remained in place 4 days for the first patient due to Foley trauma causing hematuria prior to the start of therapy but was removed in subsequent patients on the day of treatment. No other complications were observed. At no time point after treatment did pain scores exceed 1 in any patient.

Follow-up data are described in Table 3. Follow-up ranged from 8 to 24 months. For patient 1, PSA decreased from 1.96 to 1.10 ng/ml by 3 months and has remained stable up to 18 months. Patient 2 demonstrated a rise in PSA to as high as 13.32 ng/ml at 5 months and with a return to baseline by 8 months, probably representing the effects of inflammation of the prostate after therapy. PSA has been stable in patient 3 out to 12 months. Sixteen-sector mapping biopsies performed 6 months after therapy were negative for cancer in two patients with one patient (patient 3) having Gleason 6 cancer (10 %, 2 mm) in one core from the left posterior medial base. No patients developed urinary incontinence. Pre-treatment IPSS scores ranged from 2 to 20; however, scores in all patients either remained stable or decreased then stabilized. Pre-treatment erectile function according to IIEF was excellent for all patients and demonstrated no decline up to time of last follow-up.

**Table 3:** Follow-up data including PSA, biopsy results, and functional outcome

	Patient 1	Patient 2	Patient 3
Follow-up duration (months)	24	12	12
6-month 16-sector biopsy results	Negative (at 6 and 24 months)	Negative	Left posterior medial apex (10 %, 2 mm)
IPSS (baseline)	2	20	2
1 month post therapy	3	12	4
3 months post therapy	1	7	5
6 months post therapy	1	13	3
9 months post therapy	1	13	9
12 months post therapy	2	16	5
18 months post therapy	2		
IIEF (baseline) (erectile function domain/total)	30 (75)	29 (66)	30 (69)
3 months post therapy	30 (73)	30 (73)	29 (65)
6 months post therapy	30 (73)	28 (66)	30 (66)
9 months post therapy	29 (74)	30 (71)	30 (68)
12 months post therapy	30 (75)	27 (62)	30 (65)
18 months post therapy	30 (75)		

## Discussion

The present case series supports the findings of the initial experience that MRGFUS is safe and feasible for use in focal therapy of prostate cancer in humans [7, 8]. In three patients, treatment appeared to be well tolerated with minimal procedural side effects or detrimental effect to urinary or erectile function. Early biopsy data at 6 months showed reduction or no residual tumor in all patients. Biopsies remained negative at 24 months in the first patient.

The rationale behind focal therapy continues to be debated, and little evidence exists with respect to its long-term efficacy. For focal therapy to be an acceptable alternative, it is essential that the side effect profile be significantly improved compared to whole gland treatment. In a recent systematic review of focal therapy, reported continence ranged from 95 to 100 % and erectile function ranged from 54 to 100 % [9]. In an ideal state, focal therapy would provide oncologic results approaching those of the standard of care treatment with a reduction in side effects, while also providing an intervention option to mitigate anxiety related to active surveillance.

Questions remain as to which patients are best suited for focal ablative therapy. Active surveillance has assumed a prominent role in the management of indolent tumors, or those cancers that are unlikely to progress. However, some men choose active treatment even with lower risk disease due to anxiety, fear of progression, or concern about prostate cancer-related death [10]. For men diagnosed with early stage, lower risk, well-circumscribed cancers, an effective, less invasive treatment is compelling. Selection of the best candidates for focal therapy is a critical decision point. Bulky, large-volume, multifocal tumors are challenging for this technology to cover the entire targeted area or treat multiple locations which may in turn increase the need for further local treatment. Thus, patients best suited for a focal therapy procedure are those with low-risk, low-volume disease that are likely to progress or intermediate-risk disease diagnosed at a confined early stage. Questions also remain as to how much of the gland to treat, hemiablation of one lobe, or targeted ablation of just the index lesion.

Recently, an international consensus panel published guidelines on trial design for focal therapy of localized prostate cancer [11]. Ablation templates defined were targeted ablation (single target), zonal ablation, or hemiablation. Suggested inclusion criteria were Gleason 7 (3 + 4) and clinically significant Gleason 6 disease, clinical stage T1c–T2a, PSA <15 ng/ml, and life expectancy >10 years, and for patients receiving HIFU, a gland size ≤40 ml. The primary endpoint should be focal ablation of clinically significant disease, defined as prostates with a dominant tumor >0.5 ml, with negative biopsies at 12 months after treatment.

Although different treatment modalities have been evaluated for focal therapy, the modality that best optimizes cancer control and limits side effects remains unknown. MRGFUS offers several potential benefits. High-resolution MRI for prostate imaging has shown an ability to enhance anatomic evaluation and detect significant tumors and higher grade tumors. More frequently, MRI is used in active surveillance programs as an adjunctive tool to evaluate for undiagnosed lesions of higher grade or progression of disease. In the setting of radiographic-visible disease or with 3-dimensional mapping of a non-visible tumor, MRI already plays a vital role in focal therapy planning and treatment. Using the same platform for diagnosis (targeted biopsy), targeted treatment (MRGFUS), and follow-up biopsy (registered targeted biopsy) is efficient [12]. The melding of MRI and HIFU provides excellent visualization of patient anatomy, precise tumor targeting, and controlled treatment continuously monitoring the tissue effect with temperature feedback.

In a study of 42 men treated with focal standard HIFU, treatment was associated with decreases in IIEF-15 erectile function and orgasmic domains though overall IIEF-15 scores were stable and IPSS scores showed an improvement in lower urinary tract symptoms. Seventy-seven percent of the men had no evidence of cancer on 6-month biopsy [13]. In another study of 106 men treated with a variety of focal therapy modalities, 13 % experienced complications related to treatment with 2 % experiencing complications of Clavien grade 3 [14].

In the present study, a zonal ablation approach was utilized where all biopsy-positive sectors were treated. Initial results are encouraging. Functional outcome was maintained and early biopsy results were suggestive of sufficient local treatment. No patients experienced a decrement in erectile function or urinary function. Prostate biopsies at 6 months post therapy were negative in two of the three patients. In patient 3, three of the four sectors treated converted to biopsy negative and the left posterior medial base sector demonstrated a clinically insignificant amount of remaining Gleason 6 cancer (2 mm). This patient is currently being followed off therapy with plans to perform a biopsy at 24 months per protocol. Factors in patient 3 that may have contributed to persistent disease on 6-month biopsy include greater tumor burden, prior androgen deprivation therapy which may have resulted in an underestimate of tumor burden and multifocality on mapping biopsies, and marginal treatment of that sector in an attempt to avoid the adjacent urethra.

The limitations of this study to date include a small sample size and limited follow-up. The efficacy of the procedure could be limited by targeting accuracy, learning curve, or multifocality of cancer. PSA kinetics after MRGFUS are undetermined as it relates to treatment success. While the treatment time could be considered prolonged for focal treatment, the length of procedure is expected to decrease with improved technical experience.

## Conclusions

MR-guided focused ultrasound is a feasible alternative for focal therapy in a select subset of patients with prostate cancer. The treatment appears to be well tolerated with no evidence of decline in early functional outcomes. The effect on urinary control, erectile function, pain, and morbidity was minimal. While early results from a biopsy standpoint appear promising, long-term treatment efficacy requires further study.

## Abbreviations

CT: computerized tomography
DCE: dynamic contrast exam
DWI: diffusion-weighted imaging
EPI: echo planar imaging
FRFSE: fast-relaxation fast spin echo
FSE: fast spin echo
FSPGR: fast spoiled gradient echo
GRE: gradient echo
HIFU: high-intensity focused ultrasound
IIEF: International Index of Erectile Function
IPSS: International Prostate Symptom Score
IRB: Institutional Review Board
MRGFUS: magnetic resonance-guided focused ultrasound
MRI: magnetic resonance imaging
TRUS: trans-rectal ultrasound
